# Exploring the associated characteristics of Internet gaming disorder from the perspective of various game genres

**DOI:** 10.3389/fpsyt.2022.1103816

**Published:** 2023-01-12

**Authors:** Zhenjiang Liao, Xinxin Chen, Shucai Huang, Qiuping Huang, Shuhong Lin, Yifan Li, Ying Tang, Hongxian Shen

**Affiliations:** ^1^National Clinical Research Center for Mental Disorders, and Department of Psychiatry, The Second Xiangya Hospital of Central South University, Changsha, Hunan, China; ^2^Institute of Mental Health of Central South University, Chinese National Technology Institute on Mental Disorders, Hunan Key Laboratory of Psychiatry and Mental Health, Hunan Medical Center for Mental Health, Changsha, Hunan, China; ^3^Department of Psychiatry, The Fourth People’s Hospital of Wuhu, Wuhu, Anhui, China; ^4^School of Humanities and Management, Hunan University of Chinese Medicine, Changsha, Hunan, China

**Keywords:** Internet gaming disorder, gaming motivation, personality traits, game genre, gaming pattern

## Abstract

**Introduction:**

Although previous studies have reported several characteristics associated with Internet gaming disorder (IGD), the influence of game genre on IGD has rarely been investigated. This study thus aimed to compare demographic characteristics, gaming patterns, personality traits, and gaming motivations among players in different game genres, as well as identify the associated characteristics of genre-specific IGD.

**Methods:**

Internet games were classified into four types: role-playing games (RPGs), strategy (STR) games, action shooter (ACS) games, and brain and skill (BRS) games. Chinese gamers (*n* = 5,593) who usually played one of these games completed an anonymous online survey that included sociodemographic characteristics, gaming patterns, gaming motivations, the Chinese version of the Video Gaming Dependency Scale (VGD-S), and the Chinese Big Five Personality Inventory Brief Version (CBF-PI-B).

**Results:**

Significant differences were found between the genre-specific groups regarding age, gender, relationship status, VGD-S score, gaming patterns, and personality traits (e.g., RPG and STR players were more vulnerable to developing IGD compared to ACS and BRS players). Multivariate logistic regression analyses showed that the associated characteristics of IGD were slightly different within each genre-specific group after controlling for sociodemographic factors. Among them, daily gaming time and motivation for sensation seeking and escaping reality were associated with IGD development within the genre-specific group.

**Conclusion:**

Individuals in each game genre exhibited distinct characteristics that might predict IGD development (e.g., gaming patterns and personality traits). Game genre preferences should be considered in the early prevention and treatment of IGD to help high-risk individuals’ recovery. Additionally, more research should be conducted to explore RPG and STR game characteristics.

## 1. Introduction

Internet gaming disorder (IGD) was considered a tentative disorder in the fifth edition of the Diagnostic and Statistical Manual of Mental Disorders (DSM-5) (1), and it has recently been listed as a mental health disorder in the 11th edition of the International Classification of Diseases (2, 3). IGD is characterized as a persistent and compulsive pattern of game use lasting for longer than 12 months that includes impaired control and increasing priority of, and continued involvement in, gaming despite the negative consequences. Accumulated evidence has consistently shown that IGD is associated with several factors, ranging from sociodemographic variables to psychological characteristics (4–6).

Among these factors, personality traits–defined as relatively stable habitual patterns that differ across individuals and influence behaviors–might play a critical role in IGD formation (7). The five-factor model, the most accepted framework of personality traits, includes the dimensions of conscientiousness, extraversion, neuroticism, agreeableness, and openness to experience (8). These five personality traits have been found to be associated with several addictive behaviors, including smoking, drinking, and gambling (9–11). They can also be related to IGD, with higher neuroticism and decreased conscientiousness being the most consistently relevant traits (12). Gamers higher in neuroticism tend to regard reality as more threatening, which might lead them to prefer the online world, in which everything is relatively controllable and safe (13). Gamers with decreased conscientiousness are more likely to find Internet game environments attractive and to have a higher risk of developing IGD, as they are less perseverant in pursuing individual goals and paying attention to responsibilities in daily life (13). The associations between IGD and extraversion have been inconsistent in previous studies. Internet gaming offers the opportunity to communicate with other gamers, which compensates for introverted gamers’ poor social interactions in the real world (13). Nevertheless, an extraverted person may also intend to participate in gaming social networks to increase their social interactions (12).

Gaming motivation is another important factor associated with predicting IGD (14). Among these motivations, escapism–playing to avoid real-life difficulties–has been studied extensively (15), and it seems to be a common predictor of IGD among professional and non-professional gamers (16). Social interaction has also been considered an important motivation in Internet gaming (17). Other motivations (e.g., immersion) also significantly predict IGD (18, 19). Therefore, identifying gaming motivation could be crucial to understanding the differences between gamers with and without IGD, which could consequently influence the development of effective prevention and intervention measures (20).

However, although many studies have reported several characteristics associated with IGD, the impact of game genre on IGD has rarely been investigated. A previous study revealed that specific game genres may yield a higher risk of IGD, thus indicating that exploring the effects of game genre on IGD is crucial (21). Compared with other game genres, role-playing games (RPGs) have the strongest correlation with IGD (22), which can be attributed to the enticing structural characteristics arising as a result of immersing gamers in a captivating storyline (23). RPGs run continuously in real time on the Internet and possess strong sociality and competitiveness, which increase gamers’ involvement. Additionally, RPG players are more likely to experience psychological problems related to gaming (24). Further, RPG games are not the only types related to IGD. Other popular game genres, like strategy (STR) and action shooter (ACS) games, are also preferred by users with a high risk of IGD (25, 26).

Players addicted to special game genres exhibit different clinical characteristics. For example, RPG players demonstrated higher social anxiety and avoidance than other gamers (27). Additionally, addicted ACS gamers indicated significantly higher levels of trait impulsivity, disinhibition, and inattention (26). Considering the popularity of BRS games, it is necessary to include the genre to further explore the unique characteristics of IGD. Nevertheless, previous studies concentrated on the associations between specific game types and IGD without comparing the effects of different game genres on IGD. Considering the popularity and influence of Internet games, greater recognition of the clinical characteristics of different game genres could improve IGD intervention and treatment.

Previous studies rarely explored the associations between personality traits, gaming motivations, and IGD among genre-specific game users. This study thus aimed to compare the demographic characteristics, gaming patterns, personality traits, and gaming motivations among players in different game genres, as well as identify the associated characteristics of genre-specific IGD. It further explored the clinical characteristics of genre-specific users, which could ultimately lead to improved comprehension regarding IGD development in each group.

## 2. Materials and methods

### 2.1. Participants

This study involved an online survey of the Chinese population, administered from October to November 2019. Participants were recruited *via* WeChat to complete the survey on “Questionnaire Star”–a widely used online questionnaire website. The participants completed the Chinese questionnaire online by clicking the link or scanning a Quick Response code. When they first arrived at the website, they were met with a brief introduction of the study’s background, purpose, and the voluntary nature of participation, as well as assured anonymity and confidentiality. The study participants–Chinese Internet gaming users aged at least 15 years–understood the questionnaire content and agreed to participate.

### 2.2. Measures

The sociodemographic data included age, sex, and education. The Internet gaming use characteristics included game genre, monthly monetary expenditure and daily time spent on Internet gaming in the last year, and motivation for Internet gaming. The game genres were STR, ACS, BRS, and RPGs. Participants were required to choose at least one, and at most three, genres and rank them by degree of priority. The first choice was used as their most preferred genre. Gaming motivation was assessed using multiple-choice questions. Alternative answers included sensation seeking, escaping reality, coping with negative emotions, passing time, and making friends.

The Video Gaming Dependency Scale (VGDS) was used to assess IGD levels (28). The instrument was developed to cover all DSM-5 criteria, with two items pertaining to each criterion (29). The scale had 18 items rated on a four-point scale, ranging from one (strongly disagree) to four (strongly agree). Participants were required to answer each item according to their gaming behaviors over the last year. A criterion was considered to be “agreed” with if at least one of the two items was rated four (strongly agree). According to DSM-5 recommendations, participants who met five or more criteria were identified as having IGD (Cronbach’s α = 0.92).

Personality traits were assessed using the Chinese Big Five Personality Inventory Brief Version (CBF-PI-B) (30–32), which measures an individual according to the Big Five personality dimensions: conscientiousness, extraversion, agreeableness, neuroticism, and openness. The inventory contained 40 items, and each dimension was measured using eight items. Each item was scored on a six-point Likert scale, ranging from one (strongly disagree) to six (strongly agree). Previous studies have shown that the CBF-PI-B demonstrates good reliability and validity for assessing personality traits among the Chinese population (Cronbach’s α = 0.87) (32).

Since the present study was based on an online survey, the following methods were used for quality control. First, some “traps,” such as “the results of 5 plus 5,” were set in the questionnaires, and responses from those participants who answered incorrectly were excluded. Second, all the questionnaires were checked to exclude those with the same answers for ten consecutive questions, which could be considered as misfiled. Third, the average time to complete the questionnaires was measured, and those who used too much or too little time (average time ± 3SD) were excluded.

### 2.3. Statistical analysis

Differences in demographic characteristics, Internet gaming use characteristics, and CBF-PI-B scores among the four genre groups were compared and analyzed through variance (distribution of continuous variables) or chi-square (χ^2^) tests (categorical variables). A least significant difference *t*-test was used for the *post hoc* analysis. Binary logistic regression analyses were also used to identify the demographic and clinical variables related to IGD in the different genre groups. Odds ratios (ORs) and 95% confidence intervals (CIs) were calculated to quantify the associations between the variables and IGD. The significance level was set at *p* < 0.05, and all statistical analyses were performed using SPSS 23.0.

### 2.4. Ethics

The Ethics Committee of The Second Xiangya Hospital of Central South University approved the study protocol. Before completing the questionnaire, the participants had to indicate whether they refused or agreed to participate in the research. Only those willing to participate in the study could continue to answer the questionnaire.

## 3. Results

### 3.1. Sociodemographic data

Of the 5,593 participants, 3,311 were STR game users, 680 were ACS game users, 889 were BRS game users, and 713 were RPG users. The mean age of the participants in each group was 19–20 years, and the age differences were significant (*F* = 7.765, *p* < 0.001). The results of the χ^2^ test indicated significant differences regarding sex (χ^2^ = 918.553, *p* < 0.001) and relationship status (χ^2^ = 38.095, *p* < 0.001) for the four genre-specific groups. In the BRS group, the female participants outnumbered the male (female, 81.1%; male, 18.9%), but the other three groups presented a higher male proportion. This was especially true of the STR group (male, 73.6%; female, 26.4%). The results revealed that most participants in the four genre-specific groups possessed higher education levels (see Table 1).

**TABLE 1:** Sociodemographic data.

Characteristics	STR (*n* = 3311)	ACS (*n* = 680)	BRS (*n* = 889)	RPGs (*n* = 713)	*F*/χ^2^	*p*
Age	19.37 ± 1.63	19.56 ± 1.73	19.20 ± 1.44	19.50 ± 1.71	7.765	<0.001
**Gender**						
Male	2,438 (73.6%)	360 (52.9%)	168 (18.9%)	360 (50.5%)	918.553	<0.001
Female	873 (26.4%)	320 (47.1%)	721 (81.1%)	353 (49.5%)		
**Relationship status**						
Couple	62 (1.9%)	24 (3.5%)	7 (0.8%)	35 (4.9%)	38.095	<0.001
Single	3,249 (98.1%)	656 (96.5%)	882 (99.2%)	678 (95.1%)		
**Highest education**						
Lower than undergraduate	1,354 (40.9%)	264 (38.8%)	388 (43.6%)	289 (40.5%)	3.978	0.264
Undergraduate or higher	1,957 (59.1%)	416 (61.2%)	501 (56.4%)	424 (59.5%)		

STR, strategy; ACS, action shooter; RPGs, role-playing games; BRS, brain and skill.

### 3.2. Within-group comparisons of VGD-S scores, gaming patterns, personality traits, and gaming motivations

Table 2 outlines the significant differences between the genre-specific groups regarding their VGD-S scores, gaming patterns, and personality traits. *Post hoc* analyses showed that the VGD-S scores (*F* = 113.396, *p* < 0.001) and daily gaming times (*F* = 116.15, *p* < 0.001) of the participants in the RPG and STR groups were significantly higher than those of participants in the ACS and BRS groups. Further, the STR and BRS groups showed lower gaming expenditures (monthly) (*F* = 121.408, *p* < 0.001) than either the RPG or ACS groups. The RPG players evidenced higher neuroticism (*F* = 6.478, *p* < 0.001) and openness (*F* = 5.796, *p* = 0.001), but lower extraversion (*F* = 11.322, *p* = <0.001). The STR players demonstrated higher extraversion (*F* = 11.322, *p* < 0.001), but lower neuroticism, conscientiousness(*F* = 2.687, *p* = < 0.05), and openness. The ACS players demonstrated higher openness and extraversion, while the BRS users showed lower openness and extraversion and higher neuroticism and conscientiousness (see Table 2).

**TABLE 2:** Within-group comparisons of video game dependency scale scores, gaming patterns, personality traits, and gaming motivations.

	STR (*n* = 3311)	ACS (*n* = 680)	BRS (*n* = 889)	RPGs (*n* = 713)	*p*	*F*
VGD-S	2.62 (2.50)c,d	2.43 (2.66)c,d	1.08 (1.50)a,b,d	3.05 (2.85)a,b,c	<0.001	113.396
**Gaming patterns**						
Monetary expenditure on gaming/month (yuan)	60.85 (142.445)b,c,d	79.23 (154.747)a,c,d	18.17 (45.942)a,b,d	154.62 (220.367)a,b,c	<0.001	121.408
Daily gaming time (hours)	1.96 (1.25)b,c,d	1.8 (1.34)a,c,d	1.23 (0.73)a,b,d	2.37 (1.81)a,b,c	<0.001	116.15
**Personality traits**						
Neuroticism	25.31 (7.84)c,d	25.83 (8.08)	26.18 (8.11)a	26.55 (7.95)a	<0.001	6.478
Conscientiousness	32.91 (6.36)c	33.24 (6.54)	33.56 (6.12)a	32.98 (6.05)	0.045	2.687
Agreeableness	34.98 (6.06)	35.14 (6.24)	35.48 (5.76)	34.76 (6.52)	0.086	2.201
Openness	33.11 (7.06)b,d	33.93 (6.76)a,c	32.84 (7)b,d	33.92 (6.49)a,c	0.001	5.796
Extraversion	30.21 (7.16)c,d	30.43 (6.86)c,d	29.07 (6.93)a,b	28.71 (7.17)a,b	<0.001	11.322
**Gaming motivation**						
Sensation seeking	1,214 (36.7%)b,c,d	218 (32.1%)a,c,d	118 (13.3%)a,b,d	205 (28.8%)a,b,c	<0.001	180.752
Escaping	353 (10.7%)c,d	78 (11.5%)c,d	64 (7.2%)a,b,d	143 (20.1%)a,b,c	<0.001	70.178
Coping	1,545 (46.7%)c,d	299 (44.0%)c,d	260 (29.2%)a,b,d	275 (38.6%)a,b,c	<0.001	92.466
Passing time	2,190 (66.1%)c,d	445 (65.4%)c,d	676 (76%)a,b,d	429 (60.2%)a,b,c	<0.001	49.617
Making friends	727 (22.0%)c	155 (22.8%)c	59 (6.6%)a,b,d	147 (20.6%)c	<0.001	111.907

STR, strategy; ACS, action shooter; RPGs, role-playing games; BRS, brain and skill. Symbols flagging table entries denote significant differences relative to the ^a^STR, ^b^ACS, ^c^BRS, and ^d^RPG groups.

Table 2 outlines the significant differences between the genre-specific groups regarding their gaming motivations, including sensation seeking, escaping reality, coping with negative emotions, passing time, and making friends. Over 30% of the STR and ACS players reported playing games for sensation seeking (χ2 = 180.752, *p* < 0.001), while more than 20% of the RPG players reported playing games to escape reality (χ2 = 70.178, *p* < 0.001). Over 40% of the STR and ACS players indicated that they played games to cope with negative emotions. Most users (>60%) in the four groups reported playing games to pass time, and the BRS players reported the lowest motivation (6.6%) in terms of playing to make friends.

### 3.3. Identifying associated characteristics of IGD by game genre

Multivariate logistic regression analyses were conducted to identify the risk factors associated with IGD after controlling for the sociodemographic factors within each genre-specific group. These analyses revealed diverse results (see Table 3). First, greater daily gaming time (OR = 1.332; 95% CI: 1.155–1.536; *p* < 0.001), higher monthly gaming expenditure (OR = 1.004; 95% CI: 1.002–1.005; *p* < 0.001), and higher neuroticism (OR = 1.073; 95% CI: 1.036–1.112; *p* < 0.001) were associated with IGD among RPG players, in addition to lower conscientiousness (OR = 0.935; 95% CI: 0.892–0.980; *p* = 0.005), sensation seeking (OR = 2.401; 95% CI: 1.487–3.878; *p* < 0.001), and escaping reality (OR = 2.044; 95% CI: 1.157–3.612; *p* < 0.05).

**TABLE 3:** Binary logistic regression analyses of factors predicting Internet gaming disorder (IGD) in genre-specific groups.

	Variables	STR (*n* = 3311)		ACS (*n* = 680)		BRS (*n* = 889)		RPGs (*n* = 713)	
		**OR (95% Cl)**	***p***	**OR (95% Cl)**	***p***	**OR (95% Cl)**	***p***	**OR (95% Cl)**	***p***
	Gender (male vs. female)	1.524 (1.191–1.949)	0.001	1.808 (1.037–3.152)	0.037	4.464 (1.693–11.773)	0.002	1.125 (0.704–1.797)	0.624
Control variables	Education (lower than undergraduate vs. undergraduate or higher)	1.294 (1.053–1.591)	0.014	1.573 (0.898–2.755)	0.113	1.598 (0.640–3.992)	0.316	1.332 (0.806–2.202)	0.263
	**Gaming patterns**								
	Monthly monetary expenditure on gaming (yuan)	1.003 (1.002–1.003)	<0.001	1.005 (1.003–1.007)	<0.001	1.003 (0.996–1.009)	0.428	1.004 (1.002–1.005)	<0.001
	Daily gaming time (hours)	1.396 (1.294–1.506)	<0.001	1.506 (1.237–1.834)	<0.001	1.424 (1.081–1.876)	0.012	1.332 (1.155–1.536)	<0.001
	**Gaming motivations**								
Independent variables	Sensation seeking	2.360 (1.931–2.884)	<0.001	1.873 (1.099–3.190)	0.021	3.575 (1.427–8.951)	0.007	2.401 (1.487–3.878)	<0.001
	Escaping	2.321 (1.769–3.045)	<0.001	3.322 (1.691–6.527)	<0.001	4.980 (1.523–16.285)	0.008	2.044 (1.157–3.612)	0.014
	Coping	1.302 (1.067–1.589)	0.009	1.314 (0.773–2.233)	0.313	1.439 (0.568–3.648)	0.443	1.130 (0.707–1.807)	0.610
	Passing time	0.905 (0.737–1.112)	0.343	1.290 (0.744–2.236)	0.364	0.821 (0.313–2.151)	0.688	1.138 (0.706–1.835)	0.595
	Making friends	0.972 (0.771–1.226)	0.812	0.878 (0.473–1.629)	0.679	0.926 (0.240–3.569)	0.911	1.291 (0.758–2.196)	0.347
	**Personality traits**								
	Neuroticism	1.082 (1.066–1.098)	<0.001	1.062 (1.024–1.101)	0.001	1.034 (0.971–1.101)	0.251	1.073 (1.036–1.112)	<0.001
	Conscientiousness	0.918 (0.898–0.938)	<0.001	0.931 (0.881–0.984)	0.012	0.931 (0.848–1.021)	0.119	0.935 (0.892–0.980)	0.005
	Agreeableness	1.006 (0.987–1.025)	0.571	0.980 (0.933–1.030)	0.424	0.991 (0.923–1.064)	0.844	0.992 (0.954–1.032)	0.691
	Openness	1.011 (0.990–1.034)	0.301	1.002 (0.947–1.061)	0.938	0.987 (0.906–1.075)	0.755	0.977 (0.932–1.025)	0.338
	Extraversion	0.989 (0.972–1.006)	0.199	0.948 (0.906–0.992)	0.020	1.005 (0.925–1.092)	0.869	1.017 (0.980–1.055)	0.381

STR, strategy; ACS, action shooter; RPGs, role-playing games; BRS, brain and skill; OR, odd ratio.

Second, greater daily gaming time (OR = 1.369; 95% CI: 1.294–1.506; *p* < 0.001), higher monthly gaming expenditure (OR = 1.003; 95% CI: 1.002–1.003; *p* < 0.001), and higher neuroticism (OR = 1.082; 95% CI: 1.066–1.098; *p* < 0.001) were associated with IGD among STR players, in addition to lower conscientiousness (OR = 0.918; 95% CI: 0.898–0.938; *p* < 0.001), sensation seeking (OR = 2.360; 95% CI: 1.931–2.884; *p* < 0.001), escaping reality (OR = 2.321; 95% CI: 1.769–3.045; *p* < 0.001), and coping with negative emotions (OR = 1.302; 95% CI: 1.067–1.589; *p* < 0.01).

Third, greater daily gaming time (OR = 1.506; 95% CI: 1.237–1.834; *p* < 0.001), higher monthly gaming expenditure (OR = 1.005; 95% CI: 1.003–1.007; *p* < 0.001), and higher neuroticism (OR = 1.062; 95% CI: 1.024–1.101; *p* = 0.001) were associated with IGD among ACS players, in addition to lower conscientiousness (OR = 0.931; 95% CI: 0.881–0.984; *p* < 0.05), extraversion (OR = 0.948; 95% CI: 0.906–0.992; *p* < 0.05), sensation seeking (OR = 1.873; 95% CI: 1.099–3.190; *p* < 0.05), and escaping reality (OR = 3.322; 95% CI: 1.691–6.527; *p* < 0.001).

Finally, greater daily gaming time (OR = 1.424; 95% CI: 1.081–1.876; *p* < 0.05) and higher motivation for both sensation seeking (OR = 3.575; 95% CI: 1.427–8.951; *p* < 0.01) and escaping reality (OR = 4.980; 95% CI: 1.523–16.285; *p* < 0.01) were associated with IGD among the BRS players.

## 4. Discussion

To the best of the authors’ knowledge, this is not only the first study to compare personality traits and gaming motivation among users of genre-specific groups, but also the first to recognize factors related to IGD within each genre group in a large sample of Chinese adolescents and young adults. The results suggest that the users of genre-specific groups demonstrated slight differences in gaming patterns, personality traits, and gaming motivations. Further, almost identical characteristics–including daily gaming time and sensation seeking and escaping reality motivations–were associated with IGD development within the genre-specific groups.

Game genre is a critical factor that could influence IGD development; therefore, understanding the relationship between game genres and IGD development is an essential precursor for developing evidence-based theories that address the core mechanisms of IGD (33). In order to understand the related clinical characteristics, previous research has recommended identifying the preferred gaming type among IGD-suspected gamers (34). In the present study, the RPG and STR players were found to be more vulnerable to developing IGD, which aligns with the findings of previous studies. Other studies have suggested that specific genres–including RPG, FPS, and STR games–are critically associated with IGD development (26, 35). Some authors have also proposed that the highly immersive characteristics of these games and their potential online social activity functions might increase vulnerability to IGD development (27). For example, Na et al. compared the characteristics of RPG, STA, ACS, and sports games users, and identified the factors related to IGD within genre-specific groups in a large sample of South Korean adults (*n* = 5,003). RPG players showed a greater likelihood of meeting IGD criteria than did players from other game groups (34). Other studies also reported how game genres contributed to the problematic gaming patterns that underlie IGD vulnerability, especially RPG, ACS, and STR games (36). A study of 4,744 undergraduates specifically reported that STR and RPG have unequal correlations with IGD symptoms, supporting the notion that not all video games have an equivalent effect (25). Given the higher addictive potential of RPG and STR games, more research should be conducted to explore the underlying explanations of IGD from the perspective of gaming characteristics, as well as neurobiology.

Several studies have found that gaming time and monetary expenditure significantly predict IGD (28, 29). This study also identified correlations between gaming time and IGD in all groups, with RPG and STR players reporting more time spent playing games daily than the other groups. RPG and STR games are designed to engage players with realistic graphics and exciting storylines, and they do not always have a fixed end point. These combined greatly contribute to increased gaming time (37). In contrast, BRS games might yield a lower risk of developing IGD, due to their poor structural characteristics that do not inspire users to spend more time gaming. This finding supports the hypothesis that gaming-time management for STR and RPG gamers is important to prevent IGD (25). Higher monetary gaming expenditures were also considered IGD risk factors in many studies (28, 38). This study’s results revealed that ACS and RPG players spent more money per month on gaming than did the other groups, implying that suitable economic management for adolescents and young adults is needed to prevent IGD development.

Research indicates that IGD may develop more readily in complex, endless, and socially driven games, and that the vulnerabilities associated with IGD include stronger gaming motivations (22). In this study, motivation for sensation seeking and escaping reality were significantly associated with IGD in all groups. Over 30 and 40% of ACS and STR players, respectively, reported playing games for sensation seeking and coping with negative emotions. ACS and STR games operate constantly, and they feature powerful social and competitive components that entice participants to dedicate more effort to mastering game skills (21). In this process, players may experience pleasure and temporarily forget negative emotions. Over 20% of the RPG players reported a desire to escape reality. Many studies have found that the “feeling-of-escape” motivation powerfully predicts IGD (39), and that RPGs frequently involve online personas and identities that allow people to escape real-world problems (40). Further, RPGs offer players an opportunity to use their avatars to accomplish tasks that are impossible in real life, such as winning, usually in intricate stories with well-developed characters in vast immersive worlds (22).

Over 60% of users in all groups reported playing games to pass time, indicating that most players use games as an entertainment tool in a healthy way, and that only a few may develop IGD. Additionally, over 20% of the players from all groups but the BRS reported the desire to make friends through gaming. Human beings are social creatures with a need for belonging; hence, playing these games arguably offers people a novel way to fulfill these psychological needs. Given the high proportion of gamers playing to pass time (76%), a low proportion playing to make friends (6.6%), and less time spent gaming in the BRS group, it can be assumed that people play BRS games for less time and exhibit lower propensities for IGD compared with STR, ACT, and RPG players. In short, the motivations for sensation seeking and escaping reality were considered pivotal factors that influenced IGD development in the BRS group (as they presented high ORs). This suggests that, compared with the others, the players of the BRS genre have a lower propensity for IGD. Importance should also be attached to the BRS players’ motivations–particularly sensation seeking and escaping reality, due to the higher associated risk. Additionally, the BRS group had more female than male players, which could indicate a lower potential for IGD development. Researchers should also be vigilant in examining the potential risk of BRS games for female players’ IGD development.

This study’s results demonstrated that higher neuroticism and lower conscientiousness were significantly related to IGD among the STR, ACS, and RPG groups, which aligns with previous studies (41, 42). Individuals with high neuroticism often feel that the real world is dangerous; hence, they turn to virtual worlds to feel control and security (13). RPGs offer players a world in which they can experience endless winning, explore magical places, and experience intricate narratives and characters; thus, it is reasonable for RPG players to report higher neuroticism. In contrast, STR players demonstrated lower conscientiousness. One study found that STR participants exhibited high impulsivity and an impaired ability to postpone rewards (43). These two factors could partly explain the lower conscientiousness level (44). ACS players also reported higher extraversion. ACS games like *PlayerUnknown’s Battlegrounds* are known to require a high level of teamwork to win. Teammates in these games must communicate effectively and frequently; thus, the reported higher extraversion in the ACS group is reasonable. BRS players reported lower openness than players of other genres, proposing that BRS players generally retain their gaming behaviors, rather than explore new activities or other gaming genres.

This study had several limitations. First, online data based on self-reports could be biased in terms of the reliability and applicability of the conclusion. Additionally, the online survey results may have induced an artificial imbalance in the participant groups. Second, due to the study’s convenience sampling and cross-sectional design, the results’ generalizability is limited; thus, causality cannot be specifically determined. Third, the gaming industry has grown rapidly and launched new products that could not be investigated in this study (e.g., virtual reality games). Future research should be conducted to enhance the comprehension of genre-specific IGD, as well as the use of functional magnetic resonance imaging or electroencephalography to more thoroughly explore how and why IGD differs in terms of game genres. Mediating models could also be used to investigate the association between personality traits and gaming motivations to better understand the correlation between IGD and game genres. Further, more longitudinal studies should be conducted to help develop coping strategies for individuals vulnerable to IGD.

## 5. Conclusion

This study found that daily gaming time and motivation for sensation seeking and escaping reality were significantly associated with IGD within the STR, ACS, BRS, and RPG groups, suggesting that these characteristics should be paid attention to among all gaming genre groups. Further, individuals within specific game genres showed diverse characteristics–including gaming patterns, personality traits, and gaming motivations–that could predict IGD development. Therefore, the early prevention and treatment of IGD should consider the influence of different gaming genres to help high-risk individuals recover. Additionally, due to the higher risk of IGD, RPG, and STR games should be the focus of more research, and their characteristics should be explored.

## Data availability statement

The raw data supporting the conclusions of this article will be made available by the authors, without undue reservation.

## Ethics statement

The studies involving human participants were reviewed and approved by the Ethics Committee of The Second Xiangya Hospital of Central South University. The patients/participants provided their written informed consent to participate in this study.

## Author contributions

SH and QH conceptualized and designed the research. SL, YL, and YT performed the experiments. ZL and XC wrote the first draft of the manuscript. QH and HS contributed to the final manuscript. All authors approved the final manuscript.
